# Ataxia in Africa: current efforts and recommendations

**DOI:** 10.1097/MS9.0000000000003220

**Published:** 2025-04-02

**Authors:** Anano Nebieridze, Olivier Uwishema, Yusuf Jaafer Al Tarawneh, Amir I Abdelsamea, Nancy Zrara, Mugabekazi Albright Belise, Sarah Mshaymesh

**Affiliations:** a Department of Research and Education, Oli Health Magazine Organization, Kigali, Rwanda; b Faculty of Medicine, David Tvildiani Medical University, Tbilisi, Georgia; c Faculty of Medicine, RAK Medical & Health Science University, Ras Al Khaimah, United Arab Emirates; d Department of Chemistry, Faculty of Science, Benha University, Benha, Egypt; e Faculty of Medicine, Lebanese University, Beirut, Lebanon; f Faculty of Medicine and Surgery, University of Rwanda, Huye, Rwanda; g Faculty of Sciences, Haigazian University, Beirut, Lebanon

**Keywords:** africa, ataxia, coordination, mobility

## Abstract

Ataxia which is a disorder of the nervous system with the primary symptom being unsteady mobility is becoming more prevalent especially within the African continent. Nonetheless, it is suggested that there may be more cases of ataxia that are still undiagnosed roughly and this has to do with very few health care facilities offering genetic testing. This disease is most commonly associated with a variety of neurological illnesses, including stroke and brain damage leading to the uncontrollable moving of the limbs. However, in Africa, the primary contributors to the increase in ataxia are infectious diseases and food scarcity caused by drought. In our article, we analyze the different types of ataxia according to their epidemiology and describe how sociological factors aggravate the situation. We detail existing national strategies and programs aimed at solving this problem, particularly increasing funding for the infrastructure necessary for genetic testing, imagining and brain studies. Even with these measures, there remain a number of gaps with regard to epidemiology studies that need painting so as aid in accurating in the diagnosis. Awareness of the problem regarding patient education and active healthcare personnel has to be raised too. We advocate working on various fronts, including nutritional intervention, support groups for the patients, or modifying occupation therapy programs. Such measures are necessary to increase the effectiveness of ataxia treatment and improve the quality of life of patients.

## Introduction

Ataxia is a neurological condition, which is characterized by a lack of coordination in voluntary muscle movements. It can manifest as clumsiness, an unsteady gait, inability to execute complex movement, dysmetria, dysdiadochokinesis, or dyssynergia^[1,2]^. It is most commonly associated with dysfunctions of the cerebellum, but can also result from disruption in other parts of the nervous system, including the basal ganglia, cerebral cortex, spinal cord, and the sensory and vestibular systems. Based on etiology, ataxia can be classified into different types, including hereditary and acquired forms. Hereditary ataxias are often linked to genetic mutations affecting the mitochondrial functioning and DNA repair mechanisms^[3,4]^. Common hereditary forms are often associated with cerebellar atrophy, including Friedreich’s ataxia^[5]^. Acquired ataxias can result from various completely different causes like strokes, brain tumors, multiple sclerosis, alcohol abuse and nutritional deficiencies^[1]^.

**Highlights**

- Ataxia, a neurological disorder that causes unsteady mobility, is becoming increasingly prevalent in africa.
- A lack of genetic testing facilities limits early diagnosis and treatment of ataxia in the region.
- The article advocates for national strategies that include multifaceted interventions to improve ataxia diagnosis and care.

In Africa, prevalence of ataxia is determined by a plethora of factors, including, but not limited to infections, poor sanitary conditions, malnutrition, and limited access to healthcare. Illnesses like HIV, intestinal sickness, and tuberculosis (TB) are a major causes of ataxia in Africa.^[6-8]^ While ataxia is not mentioned as a direct consequence of TB, there is a strong association between CNS involvement by TB and neurological symptoms like ataxia; CNS tuberculosis, such as tuberculomas, can lead to cerebellar dysfunction, which can manifest as ataxia^[9]^. Similarly, gastrointestinal diseases can manifest as ataxia, particularly in context of celiac disease. Gluten ataxia, well-documented in medical literature, is characterised by ataxia in the presence of circulating antigliadin antibodies.^[10-12]^ Certain gastrointestinal infectious diseases can also manifest as ataxia. One such example is leptospirosis, a bacterial infection that can involve the CNS in a subset of cases. Neuroleptospirosis can present with cerebellar ataxia, likely due to immune-mediated mechanisms affecting the cerebellum^[13]^. Post-infectious cerebellar ataxia is a recognized condition, particularly in pediatric populations, where ataxia follows an infectious illness. This condition is thought to be immune-mediated^[2]^. Moreover, factors like nutritional deficiencies and exposure to harmful chemicals from the agricultural industry further exacerbate these problems^[14]^.

Diagnosis of ataxia in African populations can prove troublesome due to limited access to healthcare and unavailability of certain diagnostic tests. Hereditary ataxias, such as Friedreich’s ataxia and spinocerebellar ataxias, are present but may be less frequently diagnosed due to limited access to genetic testing^[5,15]^. While new strides have been made worldwide to make diagnosis of hereditary ataxias simpler, these innovations have yet to reach curtain regions of Africa.

Treating ataxia needs a group of diverse healthcare professionals (e.g multidisciplinary teams) and can be difficult when assets are limited^[16]^. Numerous chronic forms of ataxia do not have a specific treatment regiment, making management even more challenging^[17]^.

To improve ataxia care in Africa, we have to make strides in healthcare, by training more specialists and making certain tests and medication more available in certain regions. Approaches like physical therapy (PT) and occupational therapy (OT) are crucial to maintaining mobility and are easy to implement methods in resource limited regions. Additionally, adaptive devices, such as canes and walkers, can help improve patient independence in mobility^[16]^. Open wellcare programs and collaborative efforts from the experts worldwide are critical to successful management of ataxia in Africa^[17]^.

## Epidemiology

Similar to other parts of the word, etiology of ataxia in Africa can be subdivided into two categories: hereditary and acquired. In Africa, the epidemiology of ataxia is influenced by genetic, nutritional, and environmental factors, with distinct patterns observed in different regions.

As mentioned previously, lack of genetic testing in Africa, makes it difficult to accurately describe the incidence of certain diseases. While Friedreich Ataxia (FA) is the most commonly inherited ataxia worldwide, this disorder has been rarely reported in African populations. However, a genetically confirmed case of FA in Western Africa from Mali makes the such case. This further highlights the importance of genetic testing to improve our understanding of ataxia and provide better patient care^[18]^.

A study performed in South Africa demonstrated that autosomal dominant Spinocerebellar Ataxias (SCAs) are more prevalent, with SCA1 and SCA7 being the most common types. In families with dominant ataxia SCA1 accounted for 40.7% cases, while SCA7 for 22.2%. This is one of the highest reported frequencies globally, particularly in Black ethnic populations for SCA7^[19,20]^. The founder effect has been thought to be a major cause of high prevalence of SCA7. Identification of this pattern has greatly facilitated both targeted genetic research and therapeutic development^[20]^.

Nutritional and environmental factors also contribute to the development of ataxia. Particularly, a seasonal ataxic syndrome has been observed in Western Nigeria, primarily affecting individuals from the lower socioeconomic backgrounds, who primarily have a diet high in carbohydrates and low in protein. Seasonal Ataxia in Nigeria has been associated with consumption of *Anaphe venata* larvae and is characterized by symptoms similar to acute thiamine deficiency^[21]^. Similarly, Ataxic Syndromes have been reported in Tanganyika. These reports describe ataxic neurologic syndrome, linked to certain nutritional deficiencies, with a vast variety of symptoms, including sensory and motor ataxia, neurologic deafness and optic nerve atrophy^[22]^. Specific nutritional deficiencies like Vitamin E deficiency, can lead to neurological problems, including progressive cerebellar ataxia, due to its role as an antioxidant protecting neuronal integrity^[23,24]^. These cases underscore the impact of nutritional deficiencies and food shortages in East Africa.

## Outbreaks in Africa

The most recorded outbreak of ataxia in Africa is called Konzo. It mainly affected people in the Democratic Republic of Congo, Tanzania, and Mozambique. Konzo is a type of disease that affects the brain and nerves, causing sudden weakness and stiffness in the legs. It mostly happens in children and women of childbearing age. The problem stems from eating poorly processed cassava, which contains various cyanogenic glycosides. The spread of the outbreak usually happens at certain times of the year when food scares. During these times, people depend more on cassava for food. Poor living conditions, low socioeconomic status, lack of safe drinking water, and education on how to properly prepare food, increase the chances of konzo outbreaks^[25]^.

In the late 2000s, there was another serious case of ataxia in Nigeria. Children experienced acute balance problems after consuming seasonal fruits that were contaminated by a harmful fungus. Mycotoxins, like aflatoxins, are a big health problem in Africa because they can be found in food that is stored incorrectly^[26]^. The outbreak showed how important it is to keep food safe and to improve how we store and handle food to avoid contamination and causing health problems.

Besides these outbreaks, there have been cases of ataxia in other areas of Africa, often connected to infections like HIV. Ataxia in HIV patients is primarily associated with nervous system involvement, however, it can also result from additional mechanisms, such as direct viral effects, opportunistic infections, and immune-mediated processes. HIV can directly infect the central nervous system (CNS) and cause neurodegeneration. While exact mechanisms are yet unclear, it is thought to involve immune-mediated damage to the cerebellar neurons (e.g. Purkinje cells)^[27,28]^. HIV-related ataxia is a serious issue in Africa, due to high disease burden. Particularly, Sub-Saharan Africa accounts for approximately 70% of global HIV population^[29,30]^.

## Current efforts to mitigate Ataxia in Africa

Several studies that were conducted in multiple countries have shown an increased prevalence of all types of ataxias, where acquired cerebellar ataxia was estimated to be 27.16/100,000 in Al-Kharga district, Egypt, and 4.8/100 000 in northeastern Libya^[31,32]^. The situation is consistent across all African countries, and this requires huge efforts to mitigate ataxia by different means and in many fields, including research, diagnosis, management, etc. First, the African authorities have increased their healthcare expenditure in different sectors, including the ataxia market. as a major decision for all the subsequent strategies^[31,32]^. One of the main challenges for the treatment of ataxia is the difficulty of diagnosis and genetic testing, many researchers have emphasized this. Watson et al. have focused on spinocerebellar Ataxia type 7, which is most prevalent in South Africa^[33]^. It is an inherited polyQ disease with expanded trinucleotide CAG repeats within the coding region of the disease-causing gene, and it opens the doors for an effective allele-specific RNAi-based therapy as many clinical researchers are working on this^[34,35]^. Additionally, Cissé et al. study carried out in Mali concerning Friedreich Ataxia (FRDA), shows the multidisciplinary plan followed to diagnose and treat such a type including brain MRI, nerve conduction, blood chemistries like vitamins B12 and E, blood glucose, blood cell counts, and genetic testing by PCR and Southern blot technique^[18]^. This research also assured that the availability of genetic testing has increased in African populations, which will uncover more FRDA cases and improve therapeutic outcomes. Moreover, Osuntokun et al. has also carried out in Nigeria highlighted the importance of knowledge about vitamin deficiencies as a reason for ataxia, particularly thiamine, where it insisted on its availability and affordability in the Nigerian market and insisted on policymakers holding on to the inclusion of thiamine-rich foods in school meals^[36,37]^. Finally, even the psychosocial side was studied in a family with hereditary ataxia and concluded that it cannot be simply assumed that western-type genetic counseling is appropriate in Africa, and for that reason, an appropriate predictive service for spinocerebellar ataxia became available at Groote Schuur Hospital in South Africa^[38]^.

## Recommendations

As mentioned before, the burden of ataxia is increasing in Africa, and this requires additional guidelines with higher levels of evidence to handle the situation. Starting with the major step, healthcare expenditure in the ataxia sector should be increased, which will provide the necessary expenses for the following recommendations^[39]^. Additional epidemiological research is required and should be supported to follow up on the efficacy of the applied recommendations, besides the scientific studies that come up with improved diagnostic and therapeutic tools^[40]^. Despite the lack of disease-modifying therapies for most ataxias, many aspects of these disorders are curable; therefore, it is essential to raise awareness among healthcare professionals on the importance of a multidisciplinary approach toward ataxic patients^[41]^. Additional health professionals need to be trained to specialize in ataxia diagnosis and care. Global efforts can be made by implementing telemedicine as a way for local healthcare professionals to connect with international specialists for diagnostic support. Treatment should be highly individualized according to the patient’s symptoms, with commitment from African authorities to provide them with affordable medications and free physiotherapist and speech therapy sessions that are valuable to preserve mobility and prevent communication problems^[41]^. Hospitals should be updated on the latest diagnostic tools and financially supported by governments, to provide ataxic patients with necessary tests at fair prices, including genetic counseling like testing for Friedreich’s Ataxia through the FRDA gene, brain imaging with MRI, and blood testing for thyroid function, serum B12 and folate as primary care^[41]^. On the other hand, the treatable ataxias, which include ataxia with Vitamin E, Vitamin B12, and thiamine deficiency, can all be treated with their respective deficient vitamins. For that reason, the African markets should be supported to offer such vitamins and vitamin-fortified foods at affordable prices, and Africans should be educated on the importance of incorporating these vitamins into their diets. It’s also important to highlight the value of occupational therapy and physical therapy in maintaining patients’ independence and quality of life by providing advice on interventions like encouraging seating with support for the back and arms etc. Additionally, adaptive devices can not be overlooked as an meaningful and inexpensive way to maintain patient mobility, independence and dignity. Finally, and most importantly, it is crucial to build patient support groups where patients can meet others in the same situation, receive emotional support, and see how others cope with the symptoms, which can be of much benefit to people with Ataxia^[36]^. African nations still need to provide more financial, technological, and human capital to hasten the disease’s eradication^[42,43]^.

## Conclusion

Ultimately, tackling the rising prevalence of ataxia in Africa demands an all-encompassing, interdisciplinary approach. Government initiatives to improve healthcare facilities and diagnostic capabilities are crucial, but they should also be supported by robust epidemiological research and increased awareness. Additionally, more healthcare providers need to be trained to recognize ataxia and should have appropriate knowledge on how to manage these conditions. These results can be achieved through regional and global efforts, with incorporations on training programs and telemedicine. Implementing techniques such as food fortification, support groups for affected individuals, and vocational rehabilitation can be crucial in reducing the impact of ataxia. Through the integration of these measurements, we can improve patient outcomes, improve diagnostic accuracy, and greatly improve the quality of life for those affected by this devastating issue throughout the continent.

## Data Availability

Data availability is not applicable to this article as no new data were created or analysed in this study.
